# Long-term dietary patterns are associated with pro-inflammatory and anti-inflammatory features of the gut microbiome

**DOI:** 10.1136/gutjnl-2020-322670

**Published:** 2021-04-02

**Authors:** Laura A Bolte, Arnau Vich Vila, Floris Imhann, Valerie Collij, Ranko Gacesa, Vera Peters, Cisca Wijmenga, Alexander Kurilshikov, Marjo J E Campmans-Kuijpers, Jingyuan Fu, Gerard Dijkstra, Alexandra Zhernakova, Rinse K Weersma

**Affiliations:** 1 Department of Gastroenterology and Hepatology, University of Groningen and University Medical Centre Groningen, Groningen, The Netherlands; 2 Department of Genetics, University of Groningen and University Medical Centre Groningen, Groningen, The Netherlands; 3 Department of Pediatrics, University of Groningen and University Medical Centre Groningen, Groningen, The Netherlands

**Keywords:** diet, intestinal microbiology, meta-analysis, inflammatory bowel disease, irritable bowel syndrome

## Abstract

**Objective:**

The microbiome directly affects the balance of pro-inflammatory and anti-inflammatory responses in the gut. As microbes thrive on dietary substrates, the question arises whether we can nourish an anti-inflammatory gut ecosystem. We aim to unravel interactions between diet, gut microbiota and their functional ability to induce intestinal inflammation.

**Design:**

We investigated the relation between 173 dietary factors and the microbiome of 1425 individuals spanning four cohorts: Crohn’s disease, ulcerative colitis, irritable bowel syndrome and the general population. Shotgun metagenomic sequencing was performed to profile gut microbial composition and function. Dietary intake was assessed through food frequency questionnaires. We performed unsupervised clustering to identify dietary patterns and microbial clusters. Associations between diet and microbial features were explored per cohort, followed by a meta-analysis and heterogeneity estimation.

**Results:**

We identified 38 associations between dietary patterns and microbial clusters. Moreover, 61 individual foods and nutrients were associated with 61 species and 249 metabolic pathways in the meta-analysis across healthy individuals and patients with IBS, Crohn’s disease and UC (false discovery rate<0.05). Processed foods and animal-derived foods were consistently associated with higher abundances of Firmicutes, *Ruminococcus* species of the *Blautia* genus and endotoxin synthesis pathways. The opposite was found for plant foods and fish, which were positively associated with short-chain fatty acid-producing commensals and pathways of nutrient metabolism.

**Conclusion:**

We identified dietary patterns that consistently correlate with groups of bacteria with shared functional roles in both, health and disease. Moreover, specific foods and nutrients were associated with species known to infer mucosal protection and anti-inflammatory effects. We propose microbial mechanisms through which the diet affects inflammatory responses in the gut as a rationale for future intervention studies.

**Significance of this study**

**What is already known on this subject?**

- Western diet and low-grade intestinal inflammation are implicated in a growing number of immune-mediated inflammatory diseases.
- Diet quantity, content and timing play a major role in shaping gut microbial composition and function.
- Dysbiosis, shifts in metabolites and translocation of microbial products contribute to immune activation.
- Research has been focused on anti-inflammatory properties of isolated compounds, with limited efficacy.

**What are the new findings?**

- Diet-gut microbiome associations are consistent across patients with intestinal disease (Crohn’s disease, UC, IBS) and the general population.
- Higher intake of animal foods, processed foods, alcohol and sugar, corresponds to a microbial environment that is characteristic of inflammation, and is associated with higher levels of intestinal inflammatory markers.
- Plant-based foods are linked to short-chain fatty acid (SCFA)-producers, microbial metabolism of polysaccharides and a lower abundance of pathobionts.

**Significance of this study**

**How might it impact on clinical practice in the foreseeable future?**

- Modulation of gut microbiota through diets enriched in vegetables, legumes, grains, nuts and fish and a higher intake of plant over animal foods, has a potential to prevent intestinal inflammatory processes at the core of many chronic diseases.
- Whole food-based dietary patterns could increase the anti-inflammatory capacity of nutrients through synergistic effects on the gut microbiome.
- Sources of n-3 PUFAs (omega-3 polyunsaturated fatty acids) and polyphenols may be used to potentiate the abundance of SCFA-producers.
- Replacement of animal protein by plant protein has a potential to reduce intestinal inflammatory processes by targeting microbial pathways involved.

## Introduction

The gut microbiome directly affects the balance of pro-inflammatory and anti-inflammatory responses in the intestine. Microbial competition for nutrients plays a key role in controlling this balance.^1^ Inflammatory bowel disease (IBD) is the archetypical disease in which the homoeostasis between the gut microbiota and the intestinal immune system is lost. Beyond the local immune responses, the gut microbiota also affect systemic immune components and are implicated in a growing number of immune-mediated inflammatory diseases (IMIDs), ranging from diabetes to arthritis and systemic lupus erythematosus.^2^ Gut dysbiosis and associated inflammation have also been implicated in cancer and cardiometabolic disorders.^3 4^ Epidemiological studies uncovered several dietary factors associated with the onset of these diseases. However, the mechanisms underlying this relationship remain largely unknown.

As microbes rely on dietary substrates in the intestine, the gut microbiome is often proposed as a mediator through which foods exert their pro-inflammatory and anti-inflammatory effects. For example, animal experiments demonstrated that foods containing high levels of saturated fats,^5^ dietary heme,^6^ sugar,^7^ salt^8^ and low levels of fibre^1^ induce inflammation and autoimmunity through microbial mechanisms such as induction of T-helper 17 (TH17) cells. Other studies in mice and humans implicated that ingredients added during food processing including dietary emulsifiers,^9^ antimicrobial additives^10^ and artificial sweeteners,^11^ promote gut permeability and intestinal inflammation through an increase in mucolytic bacteria and endotoxins. In contrast, a high intake of tryptophan^12^ and fibre^13^ generally leads to immune states associated with colonic health.

The knowledge on pro-inflammatory and anti-inflammatory capacities of single compounds is increasing through functional experiments. However, there is still limited understanding of how whole foods and dietary patterns impact the gut microbiota and the host and if these impacts are different in the healthy versus the inflamed intestine. In contrast to very few food-based interventions, there have been numerous clinical trials of single nutrients. While it is easier to intervene with a pill rather than with dietary change, these trials do not acknowledge interactions of nutrients within their food matrix, which may explain the contradictory and limited effects seen.^14^ Understanding the synergies found in whole foods in the context of dietary patterns may result in more effective nutrition research and policy.

Long-term dietary interventions may be most suited for the modulation of the gut microbiota. Although extreme short-term dietary changes may still derange the gut microbiota,^15 16^ there is a tendency for microbial resilience in adults that correlates with long-term habitual diet,^17–19^ providing a constant source of dietary substrates and continuously shaping the gut ecosystem.

In this study we aimed to investigate the complex relationship between habitual diet, gut microbiota and intestinal inflammation in humans. To do so, we associated 173 dietary factors, moving from dietary patterns to specific foods and macronutrients, with the gut microbiome composition and function of 1425 individuals across four cohorts: Crohn’s disease, ulcerative colitis, irritable bowel syndrome and the general population. Analyses were performed in each cohort, followed by a meta-analysis to explore replicability of diet-microbiome associations in different disease contexts. We propose pro-inflammatory and anti-inflammatory mechanisms through which specific foods and dietary patterns could affect inflammatory responses in the gut as a rational basis for designing dietary interventions.

## Materials and methods

### Study design and cohort description

We associated the diet to the gut microbiome composition and function of 1425 individuals from the general population and patients with intestinal diseases, using two independent cohorts from the northern Netherlands. **Cohort 1** consists of 331 patients with IBD from the *1000IBD* cohort of the University Medical Center Groningen (UMCG).^20^ Patients were diagnosed by their treating physician based on accepted radiological, endoscopic and histopathological evaluation and were classified as either Crohn’s disease or ulcerative colitis. **Cohort 2** consists of 1094 individuals from the Dutch general-population-based cohort *LifeLines DEEP*.^21^ The *LifeLines DEEP* cohort comprises 223 individuals with irritable bowel syndrome (IBS) according to a symptom questionnaire based on *Rome-III* criteria. Due to known differences in the gut microbiome composition and dietary preferences between healthy individuals and patients with IBD and IBS, participants were divided into four sub-cohorts, namely Crohn’s disease (CD, n=205), ulcerative colitis (UC, n=126), IBS (n=223) and healthy controls (HC, n=871) (table 1).^22–25^ To explore consistency and heterogeneity across these different contexts, all analyses were performed separately per cohort, followed by a meta-analysis.

**Table 1:** Cohort characteristics

	CD, n=205	UC, n=124	IBS, n=223	HC, n=872
	Mean (SD)	Mean (SD)	Mean (SD)	Mean (SD)
Age	40.62 (14.19)*†	46.65 (14.83)†‡	41.44 (12.22)*‡	45.53 (13.50)
Number of males (%)	68 (33.17)†*§	60 (48.39)†‡	38 (17.04)*‡§	420 (48.17)
BMI	24.69 (4.80)*†	26.01 (4.41)†	25.30 (4.39)	25.25 (4.06)
Smokers (%)	56 (27.86)*†§	15 (12.30)*†‡	54 (24.22)§	155 (17.86)
Reads per sample	22 074 563*§ (7 406 746)	21 844 547*‡ (7 418 645)	34 408 337‡§ (12 613 562)	33 724 784 (11 388 819)
Medication use, n (%)				
ACE inhibitors	10 (4.88)*	7 (5.65)*	3 (1.35)*	38 (4.36)
AngII-receptor antagonist	3 (1.46)*	3 (2.42)*	8 (3.59)*	23 (2.64)
Ca-channel blocker	3 (1.46)*	3 (2.42)*	6 (2.69)*	15 (1.72)
Insulin	3 (1.46)*	4 (3.23)*	1 (0.45)*	3 (0.34)
Metformin	2 (0.98)*	3 (2.42)*	2 (0.90)*	11 (1.26)
Statins	8 (3.90)*	14 (11.29)*	12 (5.38)*	39 (4.47)
Macronutrients g/day				
Protein	67.09 (24.12)*	71.71 (22.03)	68.06 (16.12)*	74.79 (21.25)
Plant protein	28.52 (12.78)*	30.85 (12.08)	27.34 (7.70)*	30.79 (10.52)
Animal protein	38.65 (15.58)*	40.97 (14.57)	40.76 (11.92)*	44.05 (15.23)
Fat	76.89 (38.91)	80.66 (33.76)	71.77 (22.86)*	78.17 (29.73)
Carbohydrates	226.62 (103.8)	229.10 (91.61)	208.24 (62.75)*	228.37 (76.93)
Alcohol	4.13 (7.46)*	4.63 (6.11)*	6.60 (7.53)*	8.53 (9.39)
Total calories	1939.91 (818.23)	2010.44 (727.18)	1797.61 (475.96)*	1976.17 (621.30)
Food groups g/day				
Alcohol	55.43 (114.71)*	63.76 (91.50)*	83.42 (106.53)*	121.06 (161.63)
Breads	130.75 (87.16)	133.65 (73.03)	117.58 (54.57)*	137.43 (68.63)
Cheese	26.24 (32.64)*	28.25 (24.58)	24.96 (19.24)*	31.78 (27.65)
Dairy	228.03 (224.17)*	256.82 (185.99)	260.96 (201.40)	288.25 (194.65)
Non-alcoholic drinks	220.89 (250.73)*	157.06 (230.85)	162.67 (204.32)	144.30 (192.27)
Nuts	9.65 (17.23)*	12.25 (18.76)	12.15 (12.47)	14.46 (16.04)
Pastry	27.29 (24.74)*	33.21 (27.27)	32.47 (22.32)	31.69 (24.06)
Potatoes	83.94 (70.42)	91.67 (65.65)	66.80 (46.61)*	78.27 (52.13)
Prepared meal	45.84 (53.29)*	51.20 (72.41)*	58.16 (54.44)	56.71 (52.61)
Spreads	24.63 (29.17)	23.58 (17.90)	18.88 (14.82)*	22.33 (17.48)
Vegetables	98.02 (76.34)*	107.34 (65.78)	107.45 (58.07)	109.40 (64.54)

Mean (SD) unless stated otherwise. Cohort characteristics and food groups that significantly differed between CD, UC, IBS and controls. Full descriptive statistics can be found in online supplemental table 2. Differences in age, sex, BMI, smoking status and sequencing depth were tested between each cohort and all other cohorts. Differences in food intake and medication use were tested between each cohort and controls. χ^2^ test was performed for categorical variables and Wilcoxon-Mann-Whitney (WMW) test for continuous data.

*Significant difference compared with HC (FDR<0.05).

†Significant difference UC vs CD.

‡Significant difference UC vs IBS.

§Significant difference CD vs IBS.

AngII, angiotensin II; BMI, body mass index; Ca, calcium; CD, Crohn’s disease; FDR, false discovery rate; g, gram; HC, healthy controls; IBS, irritable bowel syndrome; UC, ulcerative colitis.

10.1136/gutjnl-2020-322670.supp2 Supplementary data

### Fecal sample collection and processing

For each participant one stool sample was collected. Participants were asked to collect and immediately freeze their sample at home. Samples were then picked up, transported on dry ice and stored at –80°C. All samples were processed according to the same pipeline in one laboratory (UMCG, Groningen). Intestinal inflammatory markers were measured in the same sample. The protocol for faecal sample collection and profiling of gut microbiota was previously published and is summarised below.^22 26^

### DNA extraction and sequencing

DNA extraction was performed using the AllPrep DNA/RNA Mini kit (Qiagen; cat. #80204) combined with mechanical lysis. Metagenomic shotgun sequencing was performed at the Broad Institute (Cambridge, Massachusetts, USA) using Illumina HiSeq platform as previously described.^22 26^ Low-quality reads were filtered out at the sequencing facility. KneadData toolkit (V.0.5.1) was used to trim the raw metagenomic reads to PHRED quality 30 and to remove Illumina adapters.

### Metagenomic profiling and filtering of samples

Reads aligning to the human genome (GRCh37/Hg19) were removed using KneadData integrated Bowtie2 tool (V.2.3.4.1), and the quality of processed metagenomes was examined using the FastQC toolkit (V.0.11.7). Functional profiles were calculated using *HUMAnN2* (V.0.10.0). The taxonomic composition was evaluated using *MetaPhlAn2* (V.2.2). Microbes and microbial functions that were present in less than 10% of samples and microbes with a relative abundance lower than 0.01% were not included in subsequent analyses. Samples with a sequencing depth below 10 million reads were removed. After filtering, 1425 samples remained for the analyses. Arcsine square-root transformations for taxonomic abundances and logarithmic transformation for pathways were used as normalisation methods. A Grubbs’ test was conducted to remove outliers. A link to the pipeline and analyses scripts is provided under the data availability. The statistical tests and terminology are further described in online supplemental table 1.

10.1136/gutjnl-2020-322670.supp1 Supplementary data

### Dietary assessment and processing of questionnaires

Dietary intake was assessed using a semi-quantitative Food Frequency Questionnaire (FFQ) that was collected concordantly with the faecal sample. The FFQ was designed and validated by the division of Human Nutrition of Wageningen University, using standardised methods.^27^ It assesses how often a food item was consumed over the previous month on a 7-item scale, along with the usual amount taken. The average daily nutrient intake was calculated by multiplying frequencies of consumption by portion size and nutrient content per gram as indicated in the Dutch Food Composition database (NEVO). Specific food items were aggregated into 25 food groups in grams per day, for example, a group of dairy composed of 21 single products such as yoghurt, buttermilk and milk^21^ (online supplemental table 2). We performed energy adjustment of the dietary intake using the nutrient density method.^28^

### Descriptive statistics

χ^2^ tests for categorical variables and Wilcoxon-Mann-Whitney test (WMW test) for continuous data were performed to calculate statistically significant differences between cohorts. Differences in age, sex, body mass index (BMI) and sequencing depth were tested between each group and all other groups. Differences in food intake and medication use were tested between each group and HCs. The gut microbiota composition of each cohort has been described previously.^22 26^

### Identification of dietary patterns and microbial clusters

Similar to microbiome data, food intake data is often zero-inflated with internal correlations of features, implying that individuals seldom eat unique foods but often consume meals with conventional food combinations.^29^ In order to identify stable dietary patterns, we performed unsupervised hierarchical clustering of the dietary intake data (in the units of gram per day) based on squared Euclidean distances. Subsequently, clustering was performed on microbial pathways using squared Euclidean distances, and on species abundance using Bray-Curtis dissimilarity as between-sample metrics. To ensure that the existence of clusters was stable and not dependent on just one set of parameters, different clustering heights and distance metrices were tested and clustering was performed in each cohort separately. A dendrogram (hierarchical tree) was visualised and clusters were defined by cutting branches off the dendrogram. As best cut, a height of 53 for the food tree, and a height of 0.8 for the species tree was identified. As the identification of clusters was stable across cohorts, clustering could ultimately be performed on the joint data set. Centroids were calculated for each participant as the mean consumption or mean abundance of all variables within a cluster.

### Association analyses per cohort

Next, we explored associations between dietary intake and microbial and pathway abundance in each cohort. For each food item or nutrient, we constructed a multivariate linear model of the food consumption adjusted for caloric intake, versus the relative abundance of taxa and pathways. Age, sex and sequencing read depth were added as covariates, represented as:

Lm: Microbial feature (taxa/pathway)~intercept + Food/nutrient + Age + Sex + Seq.depth + Cohort (HC/IBS/CD/UC)

The same model was used to test associations between dietary patterns and microbial clusters using the centroids (means) of each cluster. Since clustering was performed on the unadjusted food intake in grams per day, caloric intake (kcal) was added as a covariate:

Lm: Microbial cluster (species/pathways)~intercept + Food cluster + Age + Sex + Seq.depth + Total kcal + Cohort (HC/IBS/CD/UC)

Subsequently, dietary patterns were correlated to chromogranin A (CgA) and faecal calprotectin (Fcal) as surrogate markers for intestinal inflammation, using the same approach.

Lm: Inflammatory maker (Fcal/CgA)~intercept + Food cluster + Age + Sex + Seq.depth + Total kcal + Cohort (HC/IBS/CD/UC)

All analyses were adjusted for multiple testing using the Benjamini-Hochberg method as implemented in the *p.adjust* function in R. A false discovery rate (FDR) of <0.05 was defined as the significance cut-off.

We also explored the influence of metabolic factors, choosing phenotypes that were available in our cohort, including BMI, smoking status, hypertension (indirectly defined by the use of antihypertensives), diabetes (by the use of antidiabetics) and hyperlipidaemia (by the use of statins). The statistics thereof are provided per food-microbiome association in the online supplemental tables.

10.1136/gutjnl-2020-322670.supp3 Supplementary data

10.1136/gutjnl-2020-322670.supp4 Supplementary data

10.1136/gutjnl-2020-322670.supp5 Supplementary data

10.1136/gutjnl-2020-322670.supp6 Supplementary data

10.1136/gutjnl-2020-322670.supp7 Supplementary data

10.1136/gutjnl-2020-322670.supp8 Supplementary data

10.1136/gutjnl-2020-322670.supp9 Supplementary data

### Cross-disease meta-analysis

Next, we combined the results obtained per cohort in a meta-analysis framework in order to explore diet-microbiome relations that were significant and consistent across all cohorts. The inverse-variance method was used to calculate combined meta z-scores and corresponding meta p values. Multiple testing correction was performed per food item or food cluster for all tested taxa and pathways.

A Cochran’s Q test was conducted to measure heterogeneity between cohorts using the function *metagen* (*meta* R package (V.4.8–4)). An FDR of <0.05 and a heterogeneity p value of >0.05 in the meta-analysis were considered significant.

## Results

### Descriptive statistics

Patients with CD and individuals with IBS were younger than patients with UC and HC (mean (SD): CD 40.6 (14.2), IBS 41.4 (12.2), UC 46.65 (14.83) and HC 45.5 years of age (13.5)) (table 1). The average caloric intake was lower in IBS compared with HCs, reflecting a greater proportion of women among patients with IBS in line with the recognised 2:1 female-to-male ratio (IBS 1797.6 (476) vs HC 1976.2 kcal/day (621.3); IBS 83% vs HC 52% female). Individuals with IBS consumed less bread, potatoes, cheese, spreads and yoghurt drinks than HCs, which was reflected by a lower protein and plant protein intake (protein: IBS 68.1 (16.1) vs HC 74.8 g/day (21.3); plant protein: IBS 27.3 (7.7) vs HC 30.8 g/day (10.5)). Protein and vegetable intake was also lower in CD compared with healthy controls (protein: CD 67.1 (24.1) vs HC 74.8 g/day (21.3); group vegetables: CD 98.1 (76.3) vs HC 109.4 g/day (64.5)). Lower protein and fibre intakes in CD and IBS have been demonstrated before.^24 25^Patients with CD consumed more soft drinks than HCs as previously shown,^24^ whereas alcohol intake was higher in HCs. Cohort characteristics and differences in average food group and macronutrient intakes are given in table 1. Full descriptive statistics of all single food items are provided in online supplemental table 2. Based on this data, we corrected our analyses for age, sex, sequencing read depth and energy intake and performed analyses separately per cohort, followed by a meta-analysis and a Cochran’s Q test.

### Results of the cluster analyses

#### Unsupervised clustering identifies common dietary patterns and microbial clusters

**Figure 1:**
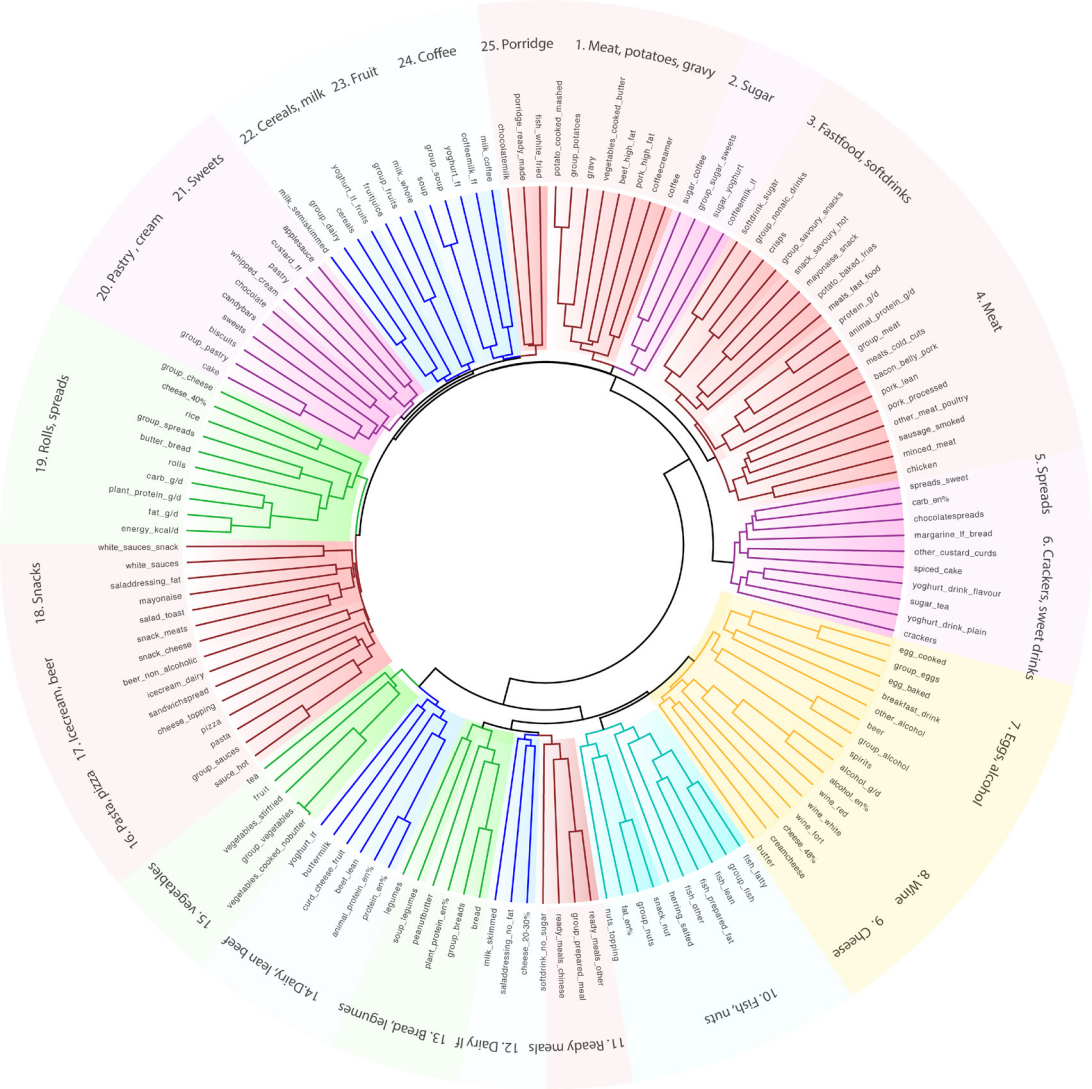
Unsupervised dietary cluster analysis reveals common food patterns. Cladogram showing clustering of the dietary intake into 25 patterns. Food frequency questionnaires were used to assess the diet of 1425 individuals comprising healthy controls (n=871), individuals with irritable bowel syndrome (n=223), Crohn’s disease (n=205) and ulcerative colitis (n=126). Unsupervised hierarchical clustering was performed using squared Euclidean distances.

Unsupervised hierarchical cluster analyses, *irrespective of disease status*, identified 25 clusters of common food pairings (figure 1). For example, cereals clustered with dairy and meat clustered with potatoes and gravy. French fries, meat, savoury snacks, mayonnaise and soft drinks formed a typical ‘fast food’ cluster.

The same analysis performed on taxonomical and functional abundances, identified 29 clusters of species with similar functions and 31 clusters of pathways of similar classes. Clusters are numbered sequentially throughout the manuscript and tables. For example, we found a cluster of commensal obligate anaerobes capable of short-chain fatty acid (SCFA) production (S1: *Bifidobacterium, Eubacterium, Dorea, Ruminococcus, Coprococcus, Subdoligranulum,* and *Faecalibacterium* spp). Another cluster was formed by *Escherichia coli, Parabacteroides* and *Bacteroides fragilis. Bifidobacterium dentium* clustered with various *Streptococcus* species that are dominant in the oral microbiome. Other clusters were composed of pathways associated with growth and survival of facultative anaerobes such as *E. coli* that have been linked to intestinal inflammation (P2: aerobic respiration, synthesis of lipopolysaccharides (LPS), heme, enterobacterial common antigen, menaquinol; P9: synthesis of enterobactin, O-antigen, quinones). The composition of clusters can be found in online supplemental table 3.

### Results of the meta-analysis

#### Meta-analysis shows similar signals across intestinal disease cohorts and controls

The meta-analysis identified significant associations between 13 dietary patterns, 24 microbial groups and markers of intestinal inflammation that were consistent across the four cohorts (FDR<0.05, p-Cochran’s-Q>0.05, online supplemental tables 4–6).

Moreover, there were 393 associations between 123 unique microbial taxa and 61 food items in the meta-analysis of individual taxa and foods (FDR<0.05, p-Cochran’s-Q>0.05, online supplemental table 7). Strikingly, 280 out of 393 results had the same direction (beta-coefficient, coef) in all cohorts, suggesting shared signals across different intestinal diseases (CD, UC and IBS) and healthy individuals. Moreover, including BMI and use of anti-diabetics, anti-hypertensives and statins as additional covariates in the model, replicated 82.2% of the results, demonstrating the robustness of the meta-analysis approach.

Plant protein, carbohydrates and red wine accounted for most associations with microbial taxa (23, 23 and 20 associations), followed by fish, nuts and animal-derived protein (17, 16 and 15 associations). Species most affected by the diet were *Lactobacillus sakei* (13), *Roseburia hominis* GCF_000225345 (12), *Faecalibacterium prausnitzii* (11), *Bifidobacterium adolescentis* (11) and *Ruminococcus obeum* (11 associations). In the same analysis, 282 pathways were related to 41 food items and nutrients. Tea, sugar used in tea, potatoes and sauces accounted for most associations with metabolic functions (48, 186, 62 and 155 associations) while they had no significant impact on microbial taxa (online supplemental table 8).

Lastly, the per-cohort analysis also showed disease-specific results that were not significant in the meta-analysis and mainly concerned species that are enriched, such as *Sutterella wadsworthensis* and *Bilophila*, or depleted in CD, UC or IBS, such as *Bifidobacterium adolescentis* (online supplemental table 7).

#### Clusters of breads, legumes, fish and nuts show a consistent negative association with several pro-inflammatory pathways

**Figure 2:**
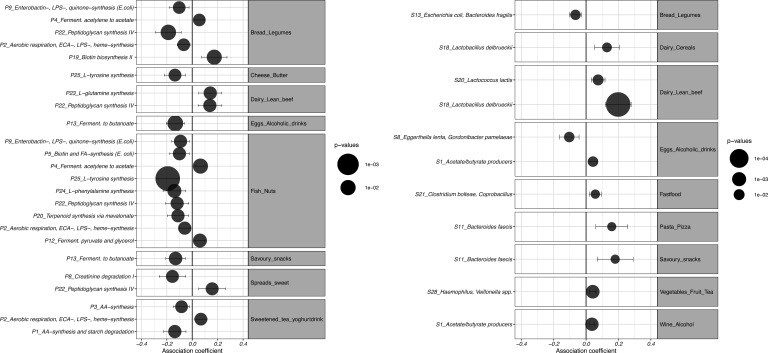
Consistent associations of dietary patterns with clusters of pathways (A) and species (B) in the cross-disease meta-analysis. Forest plot showing consistent results between dietary patterns and microbial clusters in a cross-disease meta-analysis of 1425 individuals spanning four cohorts (FDR_Meta_<0.05, p-Cochran’s-Q>0.05). Dots indicate pooled results of the meta-analysis; black lines indicate CIs. Dot size indicates the significance of the association (FDR-corrected p value). *X-axis* represents coefficients. Unsupervised hierarchical clustering was performed on dietary intake, species and pathway abundance, using squared Euclidean and Bray-Curtis distance. In each cohort, a multivariate linear model of food clusters versus microbial clusters was constructed, adding age, sex, sequencing depth and caloric intake as covariates. An inverse-variance meta-analysis was conducted on results obtained per cohort, followed by multiple testing correction and a Cochran’s Q test. AA, amino acid; ECA, enterobacterial common antigen; FA, fatty acid; FDR, false discovery rate; ferment, fermentation; LPS, lipopolysaccharides; spp, species.

A food cluster comprising breads and legumes, and a cluster of fish and nuts, were negatively associated with groups of pathways involved in the synthesis of growth factors, endotoxins and cell wall components (P2, P9, P22, online supplemental table 4, figure 2A). Moreover, we observed a negative association between the fish and nuts cluster and pathways for the synthesis of L-tyrosine, L-phenylalanine, terpenoids, quinones and fatty acids, a profile predictive of *E. coli* (P25, P24, P20, P5, figure 2A). The bread and legumes cluster was associated with a lower abundance of an *E. coli*, *Bacteroides fragilis* and *Parabacteroides* cluster (S13: FDR=0.015, coef=−0.066, online supplemental table 5, figure 2B). Conversely, the cluster of breads and legumes, and the cluster of fish and nuts, were associated with a higher abundance of pathways involved in the synthesis of acetate and the urea cycle for detoxification of ammonium (P4, figure 2A).

#### Consumption of nuts, oily fish, fruit, vegetables and cereals is linked to a higher abundance of SCFA-producers

**Figure 3:**
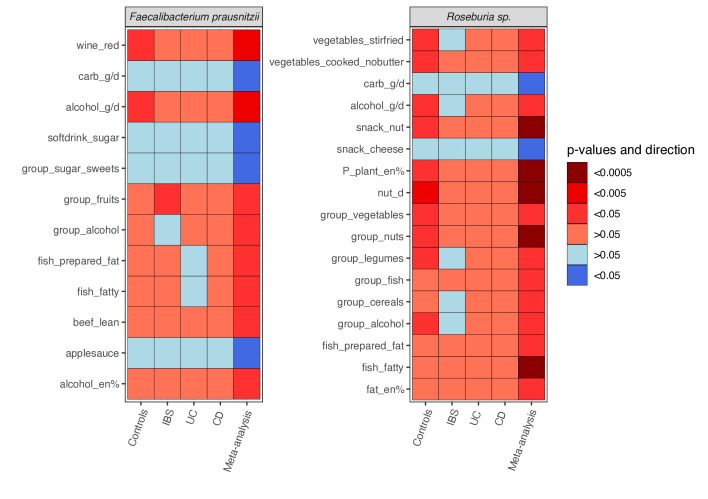
Dietary factors associated with *Faecalibacterium prausnitzii* (A) and *Roseburia* (B) relative abundance in the meta-analysis. Heatmap showing significant and consistent results of the cross-disease meta-analysis between individual foods and relative abundance of (A) *Faecalibacterium prausnitzii* and (B) *Roseburia* sp (FDR<0.05, p-Cochran’s-Q>0.05). Dietary intake was assessed by Food Frequency Questionnaires. Energy adjustment was performed by the nutrient density method. For each food item, we constructed a multivariate linear model of the food intake versus taxa and pathways, adding age, sex and sequencing depth as covariates. Association analyses were performed per cohort, followed by an inverse-variance meta-analysis, multiple testing correction and a Cochran’s Q test. carb; carbohydrates; CD, Crohn’s disease; en-%, energy-per cent; FDR, false discovery rate; g/d, gram per day; IBS, irritable bowel syndrome; nut_d, nuts added to dinner; sp, species; UC, ulcerative colitis. Red, positive association; blue, negative association. Colour density indicates significance of the association (FDR-corrected p value).

Also individually, these food items were related to several commensals capable of SCFA production (figure 3). For example, *Faecalibacterium prausnitzii* abundance was positively associated with consumption of fruits (FDR=0.005, coef=0.1), red wine (FDR=0.0003, coef=0.441) and oily fish (FDR=0.037, coef=1.695), but showed a negative association with high-sugar foods (soft drinks: FDR=0.028, coef= -0.131; sweets: FDR=0.039, coef= -0.669) (figure 3A). *Roseburia hominis* abundance was positively associated with nuts (FDR=3.80×10^–05^, coef=0.629), oily fish (FDR=0.0002, coef=1.057), vegetables (FDR=0.007, coef=0.079), legumes (FDR=0.029, coef=0.402), cereals (FDR=0.014, coef=0.485) and plant protein (FDR=1.17×10^–05^, coef=3.567) (figure 3B). These bacteria are known to have anti-inflammatory effects and provide protection of the intestinal mucosa through fermentation of fibre and pectins to acetate and butyrate.^13^ Details of each taxon and pathway, including statistics for each cohort, as well as the meta-analysis, are provided in online supplemental tables 7 and 8.

#### Red wine is associated with a higher abundance of several acetate and butyrate producers but with a lower *Bifidobacterium* abundance

A cluster of acetate and butyrate producing species (S1) was positively associated with a cluster of different types of wine (FDR=0.002, coef=0.036, figure 2B). Specifically, red wine was linked to higher abundances of *Faecalibacterium prausnitzii*, *Eubacterium hallii*, *Ruminococcus obeum*, *Ruminococcus lactaris*, *Anaerostipes hadrus* and *Alistipes putredinis* (all FDR<0.05, p-Cochran’s-Q>0.05, online supplemental table 7). Conversely, red wine intake showed a negative association with *Bifidobacterium* abundance, a SCFA-synthesising commensal (FDR=0.007, coef=−0.933).

#### Alcohol and sugar intake is associated with a higher abundance of quinone synthesis pathways

**Figure 4:**
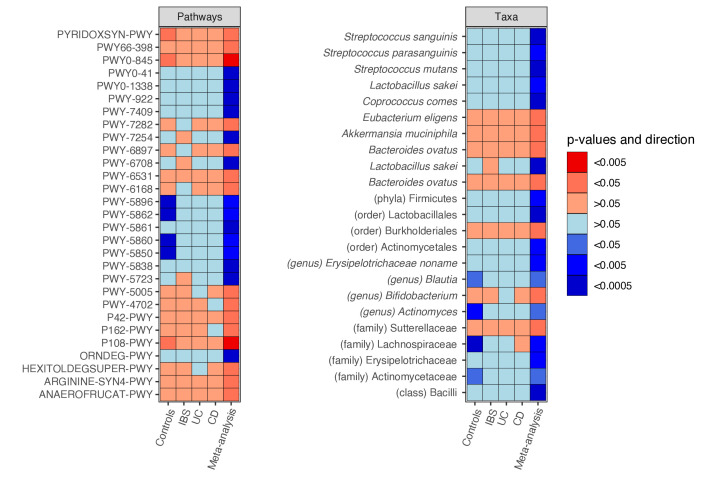
Microbial metabolic pathways (A) and taxa (B) associated with plant protein intake in the meta-analysis. Heatmap showing significant and consistent results of the cross-disease meta-analysis between plant protein intake and the relative abundance of (A) metabolic pathways and (B) taxonomical abundance of the gut microbiome (FDR<0.05, p-Cochran’s-Q>0.05). Dietary intake was assessed by Food Frequency Questionnaires. For each food item, we constructed a multivariate linear model of the food intake versus taxa and pathways, adding age, sex and sequencing depth as covariates. Association analyses were performed per cohort, followed by an inverse-variance meta-analysis, multiple testing correction and a Cochran’s Q test. CD, Crohn’s disease; FDR, false discovery rate; IBS, irritable bowel syndrome; UC, ulcerative colitis. Red, positive association; blue, negative association. Colour density indicates significance of the association (FDR-corrected p value).

Consumption of spirits (pure grain-based alcohol) was associated with a higher abundance of quinone synthesis pathways, that we previously reported to be enriched in IBD (PWY-5840, PWY-5850, PWY-5860, PWY-5862, online supplemental table 8),^22^ although after correcting for metformin use, this was not nominally significant anymore (FDR=0.094). Moreover, we observed a negative association of a pyruvate to propanoate fermentation pathway with total alcohol intake in energy-% (P108-PWY, FDR=0.0103, coef=−0.067). In contrast to alcohol and sugar, plant protein intake was negatively associated with quinone synthesis (PWY-5862, PWY-5896, online supplemental table 8, figure 4A).

#### Coffee intake is associated with a higher *Oscillibacter* abundance

Coffee consumption was significantly associated with *Oscillibacter* abundance (FDR=8.37×10^–05^, coef=0.022), heterolactic fermentation and various glycolytic pathways (PWY-6969, FUC-RHAMCAT-PWY, P122-PWY, online supplemental tables 7 and 8).

#### Lactic acid bacteria and fermentation to butanediol are consistently associated with consumption of fermented dairy

Consumption of fermented dairy like buttermilk and yoghurt showed strong associations with lactic bacteria, as previously shown,^26^ as well as with the fermentation of pyruvate to butanediol (P125-PWY) and peptidoglycan synthesis (PWY-6471) (online supplemental tables 7 and 8).

#### Plant-based food consumption is associated with higher synthesis and conversion of essential nutrients by the gut microbiota

Total intake of plant-derived protein was positively associated with pathways involved in the synthesis of SCFA (P108-PWY, P162-PWY), thiamin (PWY-6897), biotin (PWY-5005), flavin (PWY-6168), vitamin B6 (PWY0-845; PYRIDOXSYN-PWY) and L-ornithine (ARGININE-SYN4-PWY), and the degradation of sugar derivates (PWY-6531, HEXITOLDEGSUPER-PWY) (figure 4A). Participants consuming higher amounts of potatoes showed a higher abundance of starch degrading pathways (PWY-6731: FDR=0.038, coef=0.003).

#### Plant-derived and animal-derived foods and nutrients show inverse taxonomical associations

Already at the higher taxonomic levels we observed opposite relations of animal-based and plant-based foods and nutrients. While total intake of animal protein and fat was associated with a higher Firmicutes abundance, a negative association was found for plant protein and carbohydrate intake (FDR=1.30×10^-05^, coef=3.646; FDR=0.042, coef=2.936; FDR=0.003, coef=−6.081; FDR=4.67×10^-07^, coef=−1.735, respectively). Firmicutes-dominated communities have been observed in omnivores of the general population.^19^ While plant protein and bread intake were consistently linked to a higher *Bifidobacterium* abundance (FDR=0.049, coef=4.982; FDR=0.004, coef=0.815, figure 4B), total fat and animal protein intake, cheese and fish were associated with a lower *Bifidobacterium* abundance (animal protein: FDR=1.30×10^–05^, coef=−4.113), except for *Bifidobacterium dentium. Bifidobacterium dentium*, a dominant species of the upper gastrointestinal (GI) tract, showed a positive association with consumption of meat, animal protein and butter (FDR=0.001; FDR=0.048, FDR=1.91×10^–05^).

Furthermore, we observed higher abundances of Erysipelotrichaceae, *Ruminococcus* species of the Blautia genus and *Streptococcus* species with animal protein, while the opposite direction was found for plant protein intake (online supplemental table 7, figure 4B).

#### Fast food consumption is associated with higher abundances of *Blautia*, *Lachnospiraceae bacteria* and *Clostridium bolteae*

We observed significant positive associations between the consumption of fast food and savoury snacks and the abundance of *Blautia, Lachnospiraceae bacteria* and *Clostridium bolteae* in line with previous reports.^30^ A fast food cluster consisting of meats, french fries, mayonnaise and soft drinks, showed a positive association with a cluster of *Clostridium bolteae*, *Coprobacillus* and *Lachnospiraceae bacteria* (1_4_56FAA and 2_1_58FAA) (FDR=0.040, coef=0.057, figure 2B). Moreover, the cluster of fast food showed a positive association with a *Ruminococcus gnavus* and *Lachnospiraceae bacterium 1_1_57FAA* cluster in IBS and HCs, which was not significant in CD and UC in the meta-analysis (FDR_HC_=4.99×10^–5^, FDR_IBS_=3.22×10^–5^, FDR_CD_=0.490, FDR_UC_=0.761, FDR_meta_=4.69×10^–6^, coef_meta_=0.128, p-Cochran’s-Q=0.02). This finding reflects a difference in the effect size, with *Ruminococcus gnavus* being already enriched in CD and UC as compared with controls.^31^ Moreover, fast food meals and savoury snacks were positively associated with *Parabacteroides johnsonii*, *Lactobacillus sakei, Lachnospiraceae bacterium 1_1_57FAA* and the *Ruminococcus* genus across all cohorts (online supplemental table 7).

### Per cohort analysis also reveals disease-specific results

While the primary purpose of this study was to perform a meta-analysis across different conditions, heterogeneity tests and linear models performed separately per cohort also revealed disease-specific results for species that are enriched in IBD or IBS compared with controls^22 23^ (online supplemental tables 4–8). We observed a positive association of the abundance of bile tolerant bacteria such as *Sutterella wadsworthensis, Bilophila, Bacteroides* and *Alistipes* spp. with the consumption of fast food or ready meals in CD, UC and IBS. This association was not statistically significant in the HC-group in which these taxa have a lower abundance, but showed the same directionality (coefficient) (online supplemental tables 7). In IBS, consumption of buttermilk, bread and cereals was associated with a lower abundance of hydrogen producing *Dorea* spp and with a higher *Bifidobacterium* abundance (all FDR_IBS_<0.05). In UC, *Methanobacter smithii* abundance was positively associated with whole milk, butter, sauces, sweets and alcoholic drinks (all FDR_UC_<0.05). In CD, *Bacteroides vulgatus* abundance was associated with milk, animal protein and fat intake (FDR_CD_=0.002, coef =1.053; FDR_CD_=0.009, coef =4.561, FDR_CD_=0.01, coef =2.078).

### Dietary patterns are associated with intestinal inflammatory markers

Lastly, we observed significant positive associations of Fcal with the cluster comprised of fast food (FDR=4.14×10^–4^, coef=0.242) and a cluster comprised of high-fat meat, potatoes and gravy (FDR=0.003, coef=0.218), that were consistent in the meta-analysis. By contrast, we saw a negative association of Fcal with the cluster comprised of fish and nuts (FDR=0.038, coef=−0.102) and of CgA with a cluster of breads and legumes (FDR=0.005, coef=−2.484) supporting the pro-inflammatory and anti-inflammatory role of the microbial features that were associated with these foods (online supplemental tables 6).

## Discussion

In this study we have shown how habitual dietary choices can impact the human gut ecosystem and its inflammatory potential by studying the relations between unsupervised dietary patterns, intestinal inflammatory markers and gut microbial composition and function across four cohorts. We identified significant associations that replicate across patients with Crohn’s disease, ulcerative colitis, irritable bowel syndrome and the general population, implying a potential for microbiome-targeted dietary strategies to alleviate and prevent intestinal inflammation.

We showed that dietary patterns comprising legumes, breads, fish and nuts are associated with a lower abundance of clusters of opportunistic bacteria, pathways for the synthesis of endotoxins and inflammatory markers in stool. Higher proportions of these bacteria and pathways have been implicated in IBD and colorectal cancer combined with inflammation through their metabolites such as LPS.^22 32^ Conversely, we observed higher abundances of commensals such as *Roseburia, Faecalibacterium* and *Eubacterium* spp with the consumption of nuts, oily fish, fruits, vegetables, cereals and red wine across all cohorts. These bacteria are known for their anti-inflammatory effects in the intestine through fermentation of fibre to SCFAs.^13^ A dietary pattern that is traditionally high in these foods is the Mediterranean diet which has been linked to a lower IBD-risk in retrospective studies.^33^

Accumulating literature demonstrates an anti-inflammatory role of polyphenol-rich foods such as coffee, tea, red wine and fruit. We observed a higher *Oscillibacter* abundance and a lower abundance of pro-inflammatory pathways with coffee consumption. Increases in *Oscillibacter* have been shown on administration of tea-phenols or berry-phenols at the expense of potentially pathogenic species in mice.^34^ Moreover, we saw positive associations of red wine intake with several acetate and butyrate producers. Red wine polyphenols have been shown to increase *Faecalibacterium prausnitzii* and *Roseburia hominis* while reducing *E. coli* and *Enterobacter cloacae* abundance, C-reactive protein (CRP) and cholesterol levels in healthy and obese individuals.^35 36^ In contrast, total alcohol intake and spirits were associated with pro-inflammatory pathways in our study. Alcohol-induced reduction of Bifidobacteria and higher endotoxin production has been suggested to increase intestinal inflammation in patients with GI cancers and liver disease.^37^ Together, these findings support the earlier finding that moderate red wine intake is linked to higher microbial diversity, a parameter of gut health,^26 38^ while also showing that alcohol is a limiting factor, especially in the context of intestinal inflammation. Red wine polyphenol extracts may have a role to potentiate SCFA-producers and to promote beneficial actions of probiotics through a host-microbe mutualism.^35 36^

We found a consistent association of plant protein intake with several fermentation pathways and the synthesis of anti-inflammatory nutrients and L-ornithine amino acid. Concordantly, a recent study in vegetarians showed an enrichment of pathways related to carbohydrate, amino acid, cofactor and vitamin metabolism.^39^ Animal models have demonstrated that nutrients produced by microbial metabolism of plant polysaccharides downregulate the expression of pro-inflammatory cytokines,^40^ suggesting an anti-inflammatory potential of plant-based diets through gut microbial metabolism.

We consistently observed inverse taxonomical associations of animal and plant foods across all cohorts. While animal protein intake was associated with lower *Bifidobacterium* abundance, the opposite direction was found for plant protein. A lower *Bifidobacterium* abundance has been observed in omnivores compared with vegans.^15 30^ Here, we replicate this link also in patients with IBD and IBS, in whom Bifidobacteria are generally depleted. An intervention with glycated pea protein in mice has been shown to increase *Bifidobacterium* and *Lactobacillus* abundance at the expense of *Bacteroides fragilis* and *Clostridium perfringens*,^41^ suggesting a specific role of plant protein besides other plant-derived nutrients to modulate the gut microbiome.

Animal protein dominated diets also tend to include higher amounts of saturated fats, which are impactful on the microbiome themselves.^5 42^ We here observed a positive association of the total fat intake and meat consumption with species that are dominant in the upper GI tract and oral cavity, while the opposite direction was found for plant-derived foods. Higher colonisation of these bacteria in the intestine has been reported in IBD, liver cirrhosis, colon cancer^32 43^ as well as several IMIDs such as arthritis and multiple sclerosis^2^ and has been linked to high-fat diets.^30^ Microbial carbohydrate fermentation normally creates a mildly acidic environment that inhibits overgrowth of these bacteria. A switch from a normal fat/carbohydrate ratio to a high-fat diet can impact the gut microbial composition and colonic pH. While there are many disease-related factors that influence the intestinal pH, our findings tentatively suggest that a high-fat omnivore diet affects the intestinal pH, further favouring colonisation of these bacteria in the intestine, as opposed to plant-dominated diets.

By contrast, fish showed consistent positive associations with *Roseburia hominis* and *Faecalibacterium prausnitzii* in our study. Fish is high in omega-3 polyunsaturated fatty acids (n-3 PUFA). Administration of n-3 PUFA in animal models has induced a decrease in pathobionts and pro-inflammatory metabolites and increased anti-inflammatory symbionts.^5^ Conversely, high-fat diets rich in n-6 PUFAs have depleted SCFA-producers and increased CRP levels in humans.^42^ These findings imply a role for optimised n-6/n-3 fatty acid ratios for gut microbiome targeted diets.

Finally, we have shown positive associations of fast food, processed meat, soft drinks and sugar with Fcal and the abundance of *Clostridium bolteae, Ruminococcus obeum, Ruminococcus gnavus* and *Blautia hydrogenotrophica*, Firmicutes that increase energy harvesting from the diet and are implicated in obesity and IMIDs.^2 44^ Functional studies consistently demonstrated an impact of food processing on the gut microbiome, leading to gut permeability and intestinal inflammation through an increase in mucolytic bacteria like *Ruminococcus gnavus, Akkermansia muciniphila* and Proteobacteria, production of endotoxins and induction of TH17 cells.^5–11^ Especially in the combination with a low fibre intake, these bacteria turn to the mucus layer, leading to an erosion of the gut barrier.^5^ A high consumption of sugar and soft drinks combined with a low vegetable intake has already been linked to IBD.^7^ We observed higher Fcal levels with the consumption of a high-sugar and high-fat diet, while the opposite was seen for plant-based foods. While this observation may not have a clinical benefit yet in the setting of IBS or HC with pre-clinical levels of Fcal, it implies a role for dietary strategies already at the public health level. Our findings suggest the gut microbiome as a link between diet and disease risk.

Our study has several limitations related to its cross-sectional nature and the complex interplay between diet and the gut microbiome. First, the cross-sectional nature of this study cannot identify causality in the observed associations. Second, while the use of whole shot-gun metagenomic sequencing allowed us to explore predicted metabolic profiles, further studies using faecal metabolomics and in vitro studies will be needed to confirm an increase or decrease in certain microbial functions and metabolites. Third, the time that is needed to elicit a lasting response of gut microbiota to dietary changes has not been well defined. Studies suggest that long-term habitual diet has a larger impact on a ‘core’ gut microbiome composition and function^17–19^ while short-term interventions have temporary effects.^15 16 45^ Longitudinal studies using high-resolution multi-omics data and dietary interventions with long-term follow-up will help us determine the time-dynamics of the gut microbiome in future, considering day-to-day variations and intestinal transit.^29^

## Conclusions

Despite these limitations, we were able to derive dietary patterns that consistently correlate with groups of bacteria and functions known to infer mucosal protection and anti-inflammatory effects. We believe that the diet-microbiota associations that we described in this manuscript are robust: the results are consistent in the different cohorts and also remained significant after adjusting for additional cohort-specific factors such as medication usage. The findings suggest shared responses of the gut microbiota to the diet across patients with CD, UC, IBS and the general population that may be relevant to other disease contexts in which inflammation, gut microbial changes and nutrition are a common thread.^3 46^ A decrease in the here identified bacteria and their anti-inflammatory functions has already been identified in numerous inflammatory diseases, including cancer, atherosclerosis, obesity, non-alcoholic steatohepatitis, liver cirrhosis and IBD.^3 4 22 32^ Long-term diets enriched in legumes, vegetables, fruits and nuts; a higher intake of plant over animal foods with a preference for low-fat fermented dairy and fish; while avoiding strong alcoholic drinks, processed high-fat meat and soft drinks, have a potential to prevent intestinal inflammatory processes via the gut microbiome (table 2). Poor adherence to these principles has already been linked to an increased risk of IBD.^33 47 48^ We provide support for the idea that the diet can be a significant complementary therapeutic strategy through the modulation of the gut microbiome.^3 49^ For example, pre-clinical evidence shows that SCFA-producers such as *Bifidobacterium* species aid in invigorating a tumour specific T-cell response, raising the efficacy of cancer immunotherapy.^50^ It can be speculated that consumption of plant-based diets increases the abundance of these gut microbiota, further augmenting treatment responses.

**Table 2:** Overview of diet-gut microbiome associations consistent across cohorts in this study and their pro-inflammatory or anti-inflammatory role

Findings in this study			Supporting studies	
Taxa	Diet (↑)	Diet (↓)	Pro-inflammatory or anti-inflammatory role	References
*Bifidobacterium* spp	Plant protein, carbohydrates, bread, fruit	Protein, animal protein, fat, fish, savoury snacks, red wine, butter	SCFA synthesis (acetate); linked to dense mucosal barrier, reduced LPS levels and raised efficacy of cancer immunotherapy; depleted in IBD, IBS, obesity	13 15 22 35 41 51 52
*Lactococcus lactis, Lactobacillus delbrueckii*	Buttermilk, cluster of fermented dairy	No negative associations	SCFA and thiamine synthesis, anti-cancer activities	13 26 53
*Eubacterium* spp	Plant protein, cereals, fruit, red wine	Carbohydrates, non-alcoholic drinks, soft drinks	SCFA (butyrate) and phenolic acid synthesis; depleted in IBD	13 22 35
*Roseburia* spp	Fish, nuts, vegetables, plant protein, cereals, tea, legumes, vegetables, fruit	Total kcal, sugar, savoury snacks, meat, gravy, sweetened milk drinks	SCFA synthesis (butyrate) and anti-inflammatory effects; depleted in IBD	13 19 22 54
*Faecalibacterium prausnitzii*	Red wine, legumes, fruit, lean beef, fish, nuts, fat	Carbohydrates soft drinks, sweets, syrup	SCFA synthesis (butyrate) and anti-inflammatory effects; depleted in IBD	13 19 22 35 55
(phylum) Firmicutes and clusters of *Ruminococcus gnavus, Lachnospiraceae bacteria, Clostridium boltea*, *Coprobacillus*	Protein, animal protein, fat intake, cheese cluster of fast food and soft drinks	Plant protein, carbohydrates, bread	Enriched in obesity, increased energy harvesting capacity	19 44
*Bacteroides fragilis*	Cheese, custard	Cluster of breads and legumes	Opportunistic pathogen with increased abundance in IBD and colorectal cancer, raised LPS levels	22 32 41
*Escherichia coli*	No positive associations in the meta-analysis	Cluster of breads and legumes	Increased abundance in IBD and colorectal cancer, raised LPS levels	18 22 32
(family) Erysipelotrichaceae	Animal protein, soft drinks, syrup	Plant protein	Pro-inflammatory; associated with colorectal cancer, hypercholesterolaemia, and obesity.	56 57
*Streptococcus* spp	Protein, animal protein, fat, cheese, yoghurt drink, custard	Plant protein, nuts	Increased in IBD, alcoholism, liver cirrhosis, primary sclerosing cholangitis, colon cancer and IMIDs such as MS, ankylosing spondylitis and arthritis	2 22 32 43 58
*Blautia* spp	Animal protein, alcohol, meat, cheese, soft drinks, fast food pattern (*R. gnavu*s cluster)	Plant protein, carbohydrates, fruit, bread	Increased in IBD, MS, ankylosing spondylitis and arthritis	2 22 42

Diet (↑) positive relationship; Diet (↓) negative relationship.

IMIDs, immune-mediated inflammatory diseases; kcal, caloric intake; LPS, lipopolysaccharides; MS, multiple sclerosis; ref, reference; SCFA, short-chain fatty acid; spp, species.

## Data Availability

All relevant data supporting the key findings of this study are available within the article and the supplementary files. Raw metagenomic sequencing reads and extended phenotypic data are available from the European Genome-phenome Archive data repository: 1000 IBD cohort [EGAD00001004194] and LifeLines Deep cohort [EGAD00001001991]. Codes used for generating the microbial profiles are publicly available at:https://github.com/WeersmaLabIBD/Microbiome/blob/master/Protocol_metagenomic_pipeline.md]. All statistical analysis scripts are written in R and can be found here: https://github.com/WeersmaLabIBD/Microbiome/blob/master/Diet_Microbiome.md.

## References

[1] Desai MS , Seekatz AM , Koropatkin NM , et al . A dietary Fiber-Deprived gut microbiota degrades the colonic mucus barrier and enhances pathogen susceptibility. Cell 2016;167:1339–53. 10.1016/j.cell.2016.10.043 27863247 PMC5131798

[2] Forbes JD , Chen C-Y , Knox NC , et al . A comparative study of the gut microbiota in immune-mediated inflammatory diseases-does a common dysbiosis exist? Microbiome 2018;6:221. 10.1186/s40168-018-0603-4 30545401 PMC6292067

[3] Zitvogel L , Pietrocola F , Kroemer G . Nutrition, inflammation and cancer. Nat Immunol 2017;18:843–50. 10.1038/ni.3754 28722707

[4] Erridge C . Diet, commensals and the intestine as sources of pathogen-associated molecular patterns in atherosclerosis, type 2 diabetes and non-alcoholic fatty liver disease. Atherosclerosis 2011;216:1–6. 10.1016/j.atherosclerosis.2011.02.043 21439567

[5] Shen W , Gaskins HR , McIntosh MK . Influence of dietary fat on intestinal microbes, inflammation, barrier function and metabolic outcomes. J Nutr Biochem 2014;25:270–80. 10.1016/j.jnutbio.2013.09.009 24355793

[6] Constante M , Fragoso G , Calvé A , et al . Dietary heme induces gut dysbiosis, aggravates colitis, and potentiates the development of adenomas in mice. Front Microbiol 2017;8:8. 10.3389/fmicb.2017.01809 28983289 PMC5613120

[7] Racine A , Carbonnel F , Chan SSM , et al . Dietary patterns and risk of inflammatory bowel disease in Europe: results from the EPIC study. Inflamm Bowel Dis 2016;22:345–54. 10.1097/MIB.0000000000000638 26717318

[8] Wilck N , Matus MG , Kearney SM , et al . Salt-responsive gut commensal modulates T_H_17 axis and disease. Nature 2017;551:585–9. 10.1038/nature24628 29143823 PMC6070150

[9] Chassaing B , Koren O , Goodrich JK , et al . Dietary emulsifiers impact the mouse gut microbiota promoting colitis and metabolic syndrome. Nature 2015;519:92–6. 10.1038/nature14232 25731162 PMC4910713

[10] Yang H , Wang W , Romano KA , et al . A common antimicrobial additive increases colonic inflammation and colitis-associated colon tumorigenesis in mice. Sci Transl Med 2018;10. 10.1126/scitranslmed.aan4116. [Epub ahead of print: 30 05 2018]. PMC6343133 29848663

[11] Suez J , Korem T , Zeevi D , et al . Artificial sweeteners induce glucose intolerance by altering the gut microbiota. Nature 2014;514:181–6. 10.1038/nature13793 25231862

[12] Lamas B , Richard ML , Leducq V , et al . CARD9 impacts colitis by altering gut microbiota metabolism of tryptophan into aryl hydrocarbon receptor ligands. Nat Med 2016;22:598–605. 10.1038/nm.4102 27158904 PMC5087285

[13] Koh A , De Vadder F , Kovatcheva-Datchary P , et al . From dietary fiber to host physiology: short-chain fatty acids as key bacterial metabolites. Cell 2016;165:1332–45. 10.1016/j.cell.2016.05.041 27259147

[14] Forbes A , Escher J , Hébuterne X , et al . ESPEN guideline: clinical nutrition in inflammatory bowel disease. Clin Nutr 2017;36:321–47. 10.1016/j.clnu.2016.12.027 28131521

[15] David LA , Maurice CF , Carmody RN , et al . Diet rapidly and reproducibly alters the human gut microbiome. Nature 2014;505:559–63. 10.1038/nature12820 24336217 PMC3957428

[16] Klimenko NS , Tyakht AV , Popenko AS , et al . Microbiome responses to an uncontrolled short-term diet intervention in the frame of the citizen science project. Nutrients 2018;10. 10.3390/nu10050576. [Epub ahead of print: 08 May 2018]. PMC5986456 29738477

[17] Faith JJ , Guruge JL , Charbonneau M , et al . The long-term stability of the human gut microbiota. Science 2013;341:1237439. 10.1126/science.1237439 23828941 PMC3791589

[18] Wu GD , Chen J , Hoffmann C , et al . Linking long-term dietary patterns with gut microbial enterotypes. Science 2011;334:105–8. 10.1126/science.1208344 21885731 PMC3368382

[19] Wu GD , Compher C , Chen EZ , et al . Comparative metabolomics in vegans and omnivores reveal constraints on diet-dependent gut microbiota metabolite production. Gut 2016;65:63–72. 10.1136/gutjnl-2014-308209 25431456 PMC4583329

[20] Imhann F , Van der Velde KJ , Barbieri R , et al . The 1000IBD project: multi-omics data of 1000 inflammatory bowel disease patients; data release 1. BMC Gastroenterol 2019;19:5. 10.1186/s12876-018-0917-5 30621600 PMC6325838

[21] Tigchelaar EF , Zhernakova A , Dekens JAM , et al . Cohort profile: lifelines deep, a prospective, general population cohort study in the Northern Netherlands: study design and baseline characteristics. BMJ Open 2015;5:e006772. 10.1136/bmjopen-2014-006772 PMC4554905 26319774

[22] Vich Vila A , Imhann F , Collij V , et al . Gut microbiota composition and functional changes in inflammatory bowel disease and irritable bowel syndrome. Sci Transl Med 2018;10:eaap8914. 10.1126/scitranslmed.aap8914 30567928

[23] Jeffery IB , Das A , O'Herlihy E , et al . Differences in fecal Microbiomes and metabolomes of people with vs without irritable bowel syndrome and bile acid malabsorption. Gastroenterology 2020;158:1016–28. 10.1053/j.gastro.2019.11.301 31843589

[24] Peters V , Tigchelaar-Feenstra EF , Imhann F , et al . Habitual dietary intake of IBD patients differs from population controls: a case-control study. Eur J Nutr 2021;60:345–56. 10.1007/s00394-020-02250-z 32333097 PMC7867519

[25] Torres MJ , Sabate J-M , Bouchoucha M , et al . Food consumption and dietary intakes in 36,448 adults and their association with irritable bowel syndrome: Nutrinet-Santé study. Therap Adv Gastroenterol 2018;11:1756283X1774662. 10.1177/1756283X17746625 PMC5788087 29399039

[26] Zhernakova A , Kurilshikov A , Bonder MJ , et al . Population-Based metagenomics analysis reveals markers for gut microbiome composition and diversity. Science 2016;352:565–9. 10.1126/science.aad3369 27126040 PMC5240844

[27] Siebelink E , Geelen A , de Vries JHM . Self-Reported energy intake by FFQ compared with actual energy intake to maintain body weight in 516 adults. Br J Nutr 2011;106:274–81. 10.1017/S0007114511000067 21338536

[28] Willett WC , Sampson L , Stampfer MJ , et al . Reproducibility and validity of a semiquantitative food frequency questionnaire. Am J Epidemiol 1985;122:51–65. 10.1093/oxfordjournals.aje.a114086 4014201

[29] Johnson AJ , Vangay P , Al-Ghalith GA , et al . Daily sampling reveals personalized Diet-Microbiome associations in humans. Cell Host Microbe 2019;25:789–802. 10.1016/j.chom.2019.05.005 31194939

[30] Singh RK , Chang H-W , Yan D , et al . Influence of diet on the gut microbiome and implications for human health. J Transl Med 2017;15:73. 10.1186/s12967-017-1175-y 28388917 PMC5385025

[31] Hall AB , Yassour M , Sauk J , et al . A novel Ruminococcus gnavus clade enriched in inflammatory bowel disease patients. Genome Med 2017;9:103. 10.1186/s13073-017-0490-5 29183332 PMC5704459

[32] Sears CL , Garrett WS . Microbes, microbiota, and colon cancer. Cell Host Microbe 2014;15:317–28. 10.1016/j.chom.2014.02.007 24629338 PMC4003880

[33] Khalili H , Håkansson N , Chan SS , et al . Adherence to a Mediterranean diet is associated with a lower risk of later-onset Crohn's disease: results from two large prospective cohort studies. Gut 2020;69:1637–44. 10.1136/gutjnl-2019-319505 31900290

[34] Han J , Meng J , Chen S , et al . Integrative analysis of the gut microbiota and metabolome in rats treated with rice straw biochar by 16S rRNA gene sequencing and LC/MS-based metabolomics. Sci Rep 2019;9:1–9. 10.1038/s41598-019-54467-6 31780788 PMC6883064

[35] Queipo-Ortuño MI , Boto-Ordóñez M , Murri M , et al . Influence of red wine polyphenols and ethanol on the gut microbiota ecology and biochemical biomarkers. Am J Clin Nutr 2012;95:1323–34. 10.3945/ajcn.111.027847 22552027

[36] Moreno-Indias I , Sánchez-Alcoholado L , Pérez-Martínez P , et al . Red wine polyphenols modulate fecal microbiota and reduce markers of the metabolic syndrome in obese patients. Food Funct 2016;7:1775–87. 10.1039/C5FO00886G 26599039

[37] Bishehsari F , Magno E , Swanson G , et al . Alcohol and gut-derived inflammation. Alcohol Res 2017;38:163–71. 28988571 10.35946/arcr.v38.2.02 PMC5513683

[38] Le Roy CI , Wells PM , Si J , et al . Red wine consumption associated with increased gut microbiota α-diversity in 3 independent cohorts. Gastroenterology 2020;158:270-272.e2. 10.1053/j.gastro.2019.08.024 31472153

[39] De Angelis M , Ferrocino I , Calabrese FM , et al . Diet influences the functions of the human intestinal microbiome. Sci Rep 2020;10:1–15. 10.1038/s41598-020-61192-y 32144387 PMC7060259

[40] Bhat MI , Kapila R . Dietary metabolites derived from gut microbiota: critical modulators of epigenetic changes in mammals. Nutr Rev 2017;75:374–89. 10.1093/nutrit/nux001 28444216

[41] Świątecka D , Dominika Świątecka , Narbad A , et al . The study on the impact of glycated pea proteins on human intestinal bacteria. Int J Food Microbiol 2011;145:267–72. 10.1016/j.ijfoodmicro.2011.01.002 21276631

[42] Wan Y , Wang F , Yuan J , et al . Effects of dietary fat on gut microbiota and faecal metabolites, and their relationship with cardiometabolic risk factors: a 6-month randomised controlled-feeding trial. Gut 2019;68:1417–29. 10.1136/gutjnl-2018-317609 30782617

[43] Olsen I , Yamazaki K . Can oral bacteria affect the microbiome of the gut? J Oral Microbiol 2019;11:1586422. 10.1080/20002297.2019.1586422 30911359 PMC6427756

[44] Tidjani Alou M , Lagier J-C , Raoult D . Diet influence on the gut microbiota and dysbiosis related to nutritional disorders. Human Microbiome Journal 2016;1:3–11. 10.1016/j.humic.2016.09.001

[45] Maier TV , Lucio M , Lee LH , et al . Impact of dietary resistant starch on the human gut microbiome, Metaproteome, and metabolome. mBio 2017;8:e01343–17. 10.1128/mBio.01343-17 29042495 PMC5646248

[46] Lobionda S , Sittipo P , Kwon HY , et al . The role of gut microbiota in intestinal inflammation with respect to diet and extrinsic stressors. Microorganisms 2019;7:271. 10.3390/microorganisms7080271 PMC6722800 31430948

[47] Jantchou P , Morois S , Clavel-Chapelon F , et al . Animal protein intake and risk of inflammatory bowel disease: the E3N prospective study. Am J Gastroenterol 2010;105:2195–201. 10.1038/ajg.2010.192 20461067

[48] Lo C-H , Lochhead P , Khalili H , et al . Dietary inflammatory potential and risk of Crohn's disease and ulcerative colitis. Gastroenterology 2020;159:873–83. 10.1053/j.gastro.2020.05.011 32389666 PMC7502466

[49] Chiba M , Tsuji T , Komatsu M . How to optimize effects of infliximab in inflammatory bowel disease: incorporation of a plant-based diet. Gastroenterology 2020;158:1512. 10.1053/j.gastro.2019.12.050 31953070

[50] Elkrief A , Derosa L , Zitvogel L , et al . The intimate relationship between gut microbiota and cancer immunotherapy. Gut Microbes 2019;10:424–8. 10.1080/19490976.2018.1527167 30339501 PMC6546322

[51] Schroeder BO , Birchenough GMH , Ståhlman M , et al . Bifidobacteria or fiber protects against diet-induced Microbiota-Mediated colonic mucus deterioration. Cell Host Microbe 2018;23:27–40. 10.1016/j.chom.2017.11.004 29276171 PMC5764785

[52] Sivan A , Corrales L , Hubert N , et al . Commensal Bifidobacterium promotes antitumor immunity and facilitates anti-PD-L1 efficacy. Science 2015;350:1084–9. 10.1126/science.aac4255 26541606 PMC4873287

[53] Han KJ , Lee N-K , Park H , et al . Anticancer and anti-inflammatory activity of probiotic Lactococcus lactis NK34. J Microbiol Biotechnol 2015;25:1697–701. 10.4014/jmb.1503.03033 26165315

[54] De Filippis F , Pellegrini N , Vannini L , et al . High-Level adherence to a Mediterranean diet beneficially impacts the gut microbiota and associated metabolome. Gut 2016;65:1812–21. 10.1136/gutjnl-2015-309957 26416813

[55] Fava F , Gitau R , Griffin BA , et al . The type and quantity of dietary fat and carbohydrate alter faecal microbiome and short-chain fatty acid excretion in a metabolic syndrome 'at-risk' population. Int J Obes 2013;37:216–23. 10.1038/ijo.2012.33 22410962

[56] Turnbaugh PJ , Bäckhed F , Fulton L , et al . Diet-Induced obesity is linked to marked but reversible alterations in the mouse distal gut microbiome. Cell Host Microbe 2008;3:213–23. 10.1016/j.chom.2008.02.015 18407065 PMC3687783

[57] Kaakoush NO . Insights into the role of Erysipelotrichaceae in the human host. Front Cell Infect Microbiol 2015;5:84. 10.3389/fcimb.2015.00084 26636046 PMC4653637

[58] Singh RK , Chang H-W , Yan D , et al . Influence of diet on the gut microbiome and implications for human health. J Transl Med 2017;15:73. 10.1186/s12967-017-1175-y 28388917 PMC5385025

