# Supplementary material for: Long-term dietary patterns are associated with pro-inflammatory and anti-inflammatory features of the gut microbiome
Source: Gut. 2021 Apr 2;70(7):1287–98. doi: 10.1136/gutjnl-2020-322670 (PMC8223641; doi:10.1136/gutjnl-2020-322670)
Supplement: Supplementary data [file gutjnl-2020-322670supp001.pdf]

Supplementary Table 1 Description of statistical terminology and methods

Statistical method/ terminology	Description	Page
Arcsine square-root transformations	Mathematical transformation commonly used in microbiome research to transform abundances of microbial taxa (which are often left-skewed non-normally distributed) into normal-like distribution to allow for application of statistical tests that assume normality (such as Grubb's test).	7
Grubb's test	Statistical test used to detect outliers in a dataset. Assumes data can be approximated by normal distribution.	7
Zero-inflation	Dataset with a considerable proportion of zero values (for example abundance of lowly prevalent microbial taxa) is zero-inflated. Zero-inflation can bias statistical tests and necessitates use of appropriate statistical approaches that account for a large proportion of zeros (such as zero-inflated models)	7
Sequencing read depth	Total number of sequencing reads produced by the DNA sequencing.	8
Unsupervised hierarchical clustering	Data clustering method that iteratively splits data to cluster the data into a dendogram (tree structure) of clusters. Approach is unsupervised - purely data driven and does not make any prior assumptions about the data.	8
Dendogram	Diagram representing a tree, used for visualization of results of hierarchical clustering.	8
Squared Euclidean distances	Straight-line distance between two data-points, distance is equal to the length of a straight line connecting two points.	8
Bray-Curtis dissimilarity	Metric of beta-diversity, i.e. (dis)similarity of two microbiome samples. Ranges from 0 to 1, where 0 means that samples are identical and 1 that samples do not share any taxa.	8
Cluster centroids	Center of the cluster, for example data point that minimizes total within-cluster (euclidian) distance.	8
Cochran's Q test	Statistical test used to verify whether multiple treatments have identical effects, used to identify if results of a meta-analysis are dominated by a single dataset. Test is non-parametric, i.e. does not make assumptions about the underlying distribution of data, such as normality.	9
Heterogeneity p-value (p-Cochran's-Q)	P-value of Cochran's Q test - the probability of observing the test results at least as extreme as the results actually observed, under the assumption that null hypothesis is correct (that datasets contribute equally to the meta-analysis). Non-significant p-value (> 0.05) implies that null hypothesis cannot be rejected - it is unlikely that the metaanalysis is dominated by a single dataset.	9
